# The Yin and Yang of Current Antifungal Therapeutic Strategies: How Can We Harness Our Natural Defenses?

**DOI:** 10.3389/fphar.2019.00080

**Published:** 2019-02-05

**Authors:** Tomas Di Mambro, Ilaria Guerriero, Luigi Aurisicchio, Mauro Magnani, Emanuele Marra

**Affiliations:** ^1^Department of Biomolecular Sciences, University of Urbino “Carlo Bo”, Urbino, Italy; ^2^Diatheva s.r.l., Cartoceto, Italy; ^3^Takis s.r.l., Rome, Italy; ^4^Veterinary Immunotherapy and Translational Research, Rome, Italy

**Keywords:** fungal infections, fungal molecular target, monoclonal antibodies (mAB), antifungal drug development, fungal resistance

## Abstract

Fungal infections have aroused much interest over the last years because of their involvement in several human diseases. Immunocompromission due to transplant-related therapies and malignant cancer treatments are risk factors for invasive fungal infections, but also aggressive surgery, broad-spectrum antibiotics and prosthetic devices are frequently associated with infectious diseases. Current therapy is based on the administration of antifungal drugs, but the occurrence of resistant strains to the most common molecules has become a serious health-care problem. New antifungal agents are urgently needed and it is essential to identify fungal molecular targets that could offer alternatives for development of treatments. The fungal cell wall and plasma membrane are the most important structures that offer putative new targets which can be modulated in order to fight microbial infections. The development of monoclonal antibodies against new targets is a valid therapeutic strategy, both to solve resistance problems and to support the immune response, especially in immunocompromised hosts. In this review, we summarize currently used antifungal agents and propose novel therapeutic approaches, including new fungal molecular targets to be considered for drug development.

## Introduction

Infections due to pathogenic and opportunistic fungi are an ever increasing global health problem. Out of a total of about five million fungal species (spp.) worldwide, only 300 types are considered dangerous for humans, and 20–25 of these are able to affect humans on a frequent basis (Perfect, 2017). The most common yeasts isolated during clinical practices are *Candida* spp. and *Cryptococcus* spp., while *Aspergillus* spp, is the most commonly isolated filamentous fungi. Other fungi like *Fusarium* spp., *Scedosporium* spp., *Penicillium* spp., and *Zygomycetes* are also identified as being the most life-threatening species for humans (Marr et al., 2002; Husain et al., 2003). The mortality rate for invasive candidiasis is about 40% (Andes et al., 2012), while the death rate for cryptococcosis varies from 20 to 30% (Bratton et al., 2012) in wealthy countries with a fully functional health-care system. In countries where resources are limited, the death rate surpasses 50% (Nyazika et al., 2016). Instead, the mortality rate for invasive aspergillosis has diminished in the last 10 years, even if presently the plateau is steady at around 20% (Marr et al., 2015). Aggressive surgery, broad-spectrum antibiotics, prosthetic devices, grafts and general health-care associated infections increase the risk of invasive fungal infections (Enoch et al., 2006). This latter type of infection by fungal species has reached 25% of all infections contracted in hospital conditions in the past two decades. In particular, systemic infections of *Candida* have risen steadily, reaching 8–15% of all human systemic infections (Garbino et al., 2002; Eggimann et al., 2003; Hobson, 2003; Richardson, 2005).

The most widespread therapies for fungal infections are antifungal drugs, such as small molecules, monoclonal antibodies and radioimmunotherapy (RIT). At the beginning of the 2000s, RIT, a therapeutic strategy developed for cancer, was tested and tried out also for the treatment of fungal, bacterial, and viral infections, with considerable success (Dadachova et al., 2006). RIT employs the specificity of interaction between antigen and antibody to induce cytotoxicity in the target, by using radiolabeled monoclonal antibodies: this therapy was experimentally verified in the organs of mice infected systemically with *Cryptococcus neoformans* (Dadachova et al., 2003) and *Streptococcus pneumonia* (Dadachova et al., 2004). Over the past years antifungal treatments have concentrated above all on using the most common classes of small molecules and monoclonal antibodies directed against several fungal structures.

In this review, we describe both well-known and unexplored fungi molecular targets suitable for therapeutic intervention.

## Fungal Structure: a Complex System

Fungi structure is very different to that of mammalian eukaryotic cells. Fungal walls are composed of matrix components embedded and linked to scaffolds of fibrous load-bearing polysaccharides.

Most of the major structural components of fungal pathogens are not found in humans, other mammals, or plants; for this reason, the immune system of animals and plants, that represents the first defense against pathogens, have evolved to recognize many of the conserved fungal components, and many antifungal drugs have been developed to inhibit the most representative and important target molecules of fungal structure (Gow et al., 2017).

Fungal species have a double protection from the outside world: an inner plasma membrane and an outer cell wall. Structurally, the plasma membrane is a phospholipidic bilayer similar to that of all eukaryotic organisms, while the composition can vary, due to the presence of specific fungal sterols that influence membrane fluidity, such as ergosterol, which also plays an important role in plasma membrane biogenesis and function. Ergosterol is essential for the activity and distribution of integral membrane proteins, and regulation of the cell cycle (Bard et al., 1993).

Deleting genes involved in the ergosterol biosynthesis is lethal to the fungi, showing that ergosterol is crucial for fungal cell viability (Alcazar-Fuoli and Mellado, 2012). The plasma membrane is related to fungal virulence, because it is a dynamic structure that allows secretion of virulence factors, endocytosis, cell wall synthesis and invasive hyphal morphogenesis. The presence of integral membrane proteins is responsible for nutrient transport and pH sensing in the extracellular environment (Douglas and Konopka, 2016). On the basis of its specific function, the plasma membrane can be divided into domains of lipid and proteins (Martin and Arkowitz, 2014; Schuberth and Wedlich-Soldner, 2015). Several studies with *S. cerevisiae* reported that the fungal plasma membrane is organized in two domains: the most important one has been defined the Membrane Compartment of Pma1 (MCP) for the presence of the plasma membrane ATPase Pma1, and is involved in secretion and endocytosis; the other domain has been named the Membrane Compartment of Can1 (MCC) because it includes the arginine transporter Can1 (Grossmann et al., 2007; Malinsky et al., 2010; Olivera-Couto et al., 2011; Murphy and Kim, 2012; Douglas and Konopka, 2014). Two peripheral membrane proteins, Pil1 and Lsp1, bind the MCC at the cytoplasmic portion, forming a complex named eisosome, from the fusion of the two Greek terms “eis” (into or portal) and “soma” (body), because they were described as the sites of endocytosis (Walther et al., 2006). Successive studies have discredited this hypothesis, suggesting that MCC/eisosomes function as an area of plasma membrane which protects proteins against internalization: this is an essential regulatory mechanism, with regard to specific transporters, in order to ensure their stability in the membrane (Grossmann et al., 2008).

In addition to the plasma membrane, the most external envelope is the fungal cell wall, that constitutes the first defense against hostile environmental conditions. The dynamism of this structure is essential not only for cell viability and morphology, but also for fungal virulence; for this reason, the cell wall is the site of several signaling pathways which are activated, interacting with each other, under a finely regulated mechanism. The first evidence of the existence of signaling pathways at the cell wall was described in *Saccharomyces cerevisiae*, which resulted as a simple fungal model for molecular studies, due to its unicellular organization (Levin, 2011). In the last decades, different pathways have been identified as common and conserved across fungal species, such as mitogen activated protein kinases (MAPKs), calcineurin, cAMP, the target of rapamycin (Tor), and the entirety of these cascades is known as the Cell Wall Integrity pathway (Grosse et al., 2008; Rispail et al., 2009; Baldin et al., 2015). All the stress signals received at the cell surface receptors are sent to the intracellular portion through downstream effectors that activate the production of cell wall components. The cell wall is generally organized as a scaffold of carbohydrate polymers to which a variety of proteins and other components are added, creating a strong but elastic structure (Gow et al., 2017). The polysaccharide composition can vary among the different species, but is characterized by conserved parts, such as a core of branched β-1,3-glucan-chitin (Valiante et al., 2015). Chitin is produced as linear chains of β-1,4 *N*-acetylglucosamine and constitutes the most primordial structural polysaccharide in the fungal cell wall. Many families of enzymes with the role of chitin synthesis have been identified *in silico*, but the precise biochemical functions of many chitin synthase isoforms are unknown (Roncero, 2002). A glucan synthase complex associated to the plasma membrane is responsible for the β-1,3 glucan synthesis: by using UDP-glucose as a substrate, linear β-1,3 glucan chains are extruded through the membrane into the cell wall for the subsequent transglycosylation (Douglas, 2001). This protein complex is constituted by a Rho1 regulatory subunit and a Fks catalytic subunit, which is an integral plasma membrane protein with up to 16 *trans*-membrane helices. β-1,3 glucan chains are very abundant and important for cell wall maintenance and virulence function.

## Synthetic Small Molecules: an Overview of Antifungal Drugs and Improvements

**FIGURE 1:**
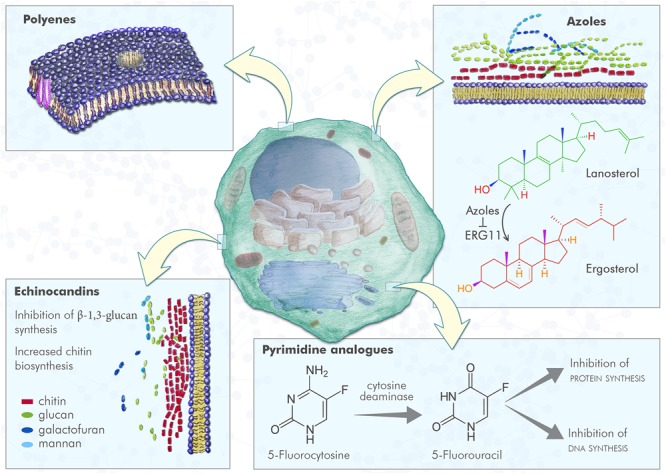
Fungal cell and antifungal mechanism of action. Polyenes induce the formation of pores in fungal plasma membrane, with a consequent increased membrane permeability; azoles inhibit ERG11, the enzyme that converts lanosterol in 4,4-dimethylcholesta-8,14,24-trienol that can be subsequently converted to ergosterol, leading to lack of ergosterol and cell toxicity due to lanosterol accumulation; pyrimidine analogs are compounds incorporated into fungal RNA, that interfere with fungal RNA and DNA metabolism; echinocandins inhibit the β-1,3 glucan synthesis resulting in an increased chitin biosynthesis.

Antifungal drugs currently used can be divided in four major classes, on the basis of their targets and mechanism of action (Table 1 and Figure 1):

**Table 1:** Current antifungal drugs described through their mechanism of action and biological effect.

Antifungal drug family	Mechanism of action	Biological effect
Polyenes	Formation of pores in the fungal cell membrane	Increased membrane permeability accumulation of toxic ROS
Pyrimidine analogs	Interfering with the fungal RNA and DNA metabolism	Impairment of the fungal RNA and DNA synthesis
Azoles	Inhibition of the fungal enzyme Erg11	Block of the lanosterol to ergosterol conversion
Echinocandins	Inhibition of the fungal enzyme β1,3-glucan synthase	Block of the cell wall β1,3-glucan synthesis

• **polyenes**: amphotericin B is a small molecule and was the first antifungal drug approved. This drug acts by binding irreversibly to ergosterol-containing fungal membrane producing a channel that changes the membrane permeability with the emission of intracellular components^1^;
• **pyrimidine analogs**: flucytosine is a drug that through the cytosine permease is able to enter fungal cells, where it is converted into 5-fluorouracil by a cytosine deaminase. This latter compound is incorporated into fungal RNA, interfering with the RNA and DNA metabolism of fungi (see footnote 1);
• **azoles**: small molecules that belong to this family work by inhibiting the lanosterol 14α-demethylase (Erg11), the enzyme that converts lanosterol to ergosterol through forming the intermediate compound 4,4-dimethylcholesta-8,14,24-trienol, and consequently blocking ergosterol synthesis. From the first generation of azoles in the late 1970s, to the third one in the first decade of the 2000s, activity, safety, pharmacokinetics and new formulations have been improved.
• **echinocandins**: these antifungal drugs act by blocking the β (1,3)-D-glucan synthase, an enzyme that is essential for synthesis of β-1,3 glucan, a principal component of the cell wall of fungi that is not present in mammalian cells.

Fungal species have developed different mechanisms of resistance to antifungal drugs.

Resistance to amphotericin B is due to point mutations in the ergosterol biosynthetic pathway (Dick et al., 1980; Karyotakis et al., 1993; White et al., 1998; Ghannoum and Rice, 1999).

Mutations in the Fur1 gene (uracil phosphoribosyl transferase) and in the Fcy1 gene (cytosine deaminase), decrease conversion of the prodrug flucytosine into its active-toxic form 5-fluorouracil (Vanden Bossche et al., 1994; Edlind and Katiyar, 2010). The azoles family in *C. albicans*, reports three mechanisms of resistance: the reduction of azole accumulation through active efflux, the alteration or overexpression of the binding site (14α-sterol-demethylase, encoded by *ERG11*) and the loss-of-function downstream mutation in the ergosterol pathway (defective Δ-5,6-desaturase encoded by *ERG3*) (Karyotakis et al., 1993; Kontoyiannis and Lewis, 2002). Echinocandins resistance has grown with the increasing use of these drugs in hospitals.

This resistance form is related to mutations in FKS1 which encodes the 1,3-β-D-glucan enzyme that concurs in the formation of the fungal cell wall. This mechanism has been most prominently observed in the haploid yeast, *C. glabrata*, and has recently been observed in mutations on MSH1 (Alexander et al., 2013; Perfect, 2017).

To overcome this hurdle, researchers have improved the present antifungal drugs and, on the basis of their categorization by type of compound, it is possible to focus on the different chemical modifications that can make antifungal drugs therapeutic also against fungal resistant strains:

• the improvements in **polyenes** are based on a modification in the drug structure that transforms the molecular-umbrella-conjugates to nanoparticles and polysaccharide-conjugates. The development of amphotericin B cochleate lipid-crystal nanoparticles as oral drug, showed *in vivo* activity and it is undergoing clinical trials (NCT02733432 and NCT02629419) (Delmas et al., 2002);
• the improvements in **echinocandins** consider two different modifications: the first is based on a chemical change of the backbone to increase the stability, such as for CD101, whose clinical trials entered into phase II (NCT02733432 and NCT02734862) (Ong et al., 2016; Pfaller et al., 2016); the second concerns the 1,3-β glucan synthase inhibitor SCY078 (formerly MK-3118) developed by Scynexis and currently in phase II (NCT02679456). This specific drug is a triterpene and has an anti-yeast activity similar to echinocandins and, according to *in vitro* tests, is effective even against echinocandin-resistant yeasts (Walker et al., 2011);
• the improvements in **pyrimidine analogs** have produced a new compound, named F901318, that is able to inhibit the fungal dehydroorotate dehydrogenase, which is crucially involved in fungal pyrimidine biosynthesis (Oliver et al., 2016; Du Pre et al., 2018);
• the improvements in **azoles** have been carried out by Viamet Pharmaceutical and are based on chemical modifications able to manipulate the metal-binding groups of azole compounds, reducing their interaction with cytochrome P450 and thus fewer potential drug-drug interactions. There are three new compounds:
  (1) VT-1161, now in phase II of clinical trials (NCT02267356 and NCT02267382) and developed to fight onychomycosis and vaginal candidiasis (Warrilow et al., 2014; Gebremariam et al., 2015);
  (2) VT-1129, aimed toward cryptococcosis, worked exceptionally *in vitro* and in animal models;
  (3) VT-1598 is powerful and works against endemic mycosis and cryptococcosis (Lockhart et al., 2016).

## Monoclonal Antibodies: New Biological Antifungal Drugs

Monoclonal antibodies (mAbs) represent one of the most powerful therapeutic and diagnostic tools of modern medicine. Characteristics like specificity against antigens, high reproducibility and a high degree of purity has made these molecules extremely promising therapeutic agents in different clinic contexts such as cancer, infective and autoimmune diseases.

Upon immunization with the target antigen, transgenic mice produce specific human antibodies against the antigen and can be used to clone human antibodies with the conventional hybridoma technique. The advantage of this approach is the possibility to generate *in vivo* matured antibodies and the expression of full-length IgG. However, this specific method shows some disadvantages such as the use of murine-toxic antigens and the high similarity of the target antigen to its murine homologs. Since the 1990s, with the development of chimeric and humanized monoclonal antibodies, this drug class has been the most rapidly growing and advancing. Transgenic humanized mice provide an inestimable instrument for entirely human antibody production, and were created by replacing the mouse antibody gene with a human Ig loci (Lonberg, 2008). Recently, a new approach called CDR-grafting, generates improved humanized antibodies, which present a 85–90% content of human origins, and result less immunogenic than human-mouse chimeric (Clavero-Alvarez et al., 2018).

During the last 8 years monoclonal antibody therapy in late stage clinical studies has increased from 20 newly produced antibodies to over 60, with a global market of US$ 86 billion in 2016 and with an estimated increase over the next 4 years of US$ 39 billion ($125 billion) (Gaughan, 2016; Reichert, 2017; Carter and Lazar, 2018; Kaplon and Reichert, 2018).

In the field of fungal infectious diseases, several mAbs have been produced (Table 2) and some examples are listed below.

**Table 2:** Antifungal mAbs described through the pathogen and the specific antigen that are recognized.

Antifungal mAbs	Source	Pathogen	Antigen
mAb C7	Mouse	*C. albicans, C. lusitaniae, C. neoformans, A. fumigatus, S. prolificans*	Cell wall mannoprotein
mAb A9	Mouse	*A. fumigatus*	Cell wall glycoprotein
mAb 7B8 and 8G4	Mouse	*A. fumigatus* and *flavus*	Galactomannan of *A. fumigatus*
mAb 18B7	Mouse	*Cryptococcus* spp.	Glucuronoxylomannan
Mycograb	Human	*Candida* spp.	Candida HSP(90)

### MAb C7

In order to fight candidiasis, a monoclonal antibody directed against a *C. albicans* cell wall mannoprotein was developed, demonstrating its ability to inhibit fungal adhesion and germination, and indicating a direct candidacidal activity (Moragues et al., 2003). Antibodies can reduce the adhesion of *C. albicans* to host surfaces by blocking the fungal cell wall adhesins (San Millan et al., 2000) and also decreasing germination, another important activity involved in the adhesion mechanism (Kimura and Pearsall, 1980). MAb C7 was tested in murine models of invasive candidiasis and demonstrated protection against *C. albicans* infection with both a longer mean survival time and a higher percentage of final survival (Sevilla et al., 2006). Further experiments have shown that mAb C7 was able to inhibit the growth of other fungal species, such as *Candida lusitaniae, Cryptococcus neoformans, A. fumigatus*, and *Scedosporium prolificans* (Moragues et al., 2003). MAb C7 also exerted a candidacidal effect by impairing the iron uptake mechanism of *C. albicans*. In fact, all the fungal genes that showed deregulated expression after MAb C7 treatment were involved in iron uptake and homeostasis (Brena et al., 2011); furthermore, mAb C7 can also recognize Als3p, a hyphal-specific glycophosphatidylinositol cell wall protein that is responsible for interaction with host epithelial cells to acquire iron from their ferritin (Sui et al., 2017). All these data can explain the multiple action mechanisms of this monoclonal antibody.

### MAb A9

During these same years, another monoclonal antibody was produced by using cell wall glycoprotein of *A. fumigatus* as antigen for mice immunization. MAb A9 was able to reduce hyphal development and the duration of spore germination, as demonstrated in proliferation *in vitro* assays. The efficacy of mAb A9 was also tested in murine models of invasive aspergillosis, showing the capacity to reduce fungal growth in specific organs, such as the kidney, and to increase the survival rate of treated animals compared to their negative controls (Chaturvedi et al., 2005).

### MAbs 7B8 and 8G4

A very recent study reported novel murine mAbs that recognized *A. fumigatus* galactomannan. Mice were immunized with a synthetic pentasaccharide β-D-Gal*f*-(1,5)-[β-D-Gal*f*-(1,5)]_3_-α-D-Man*p*, structurally related to *A. fumigatus* galactomannan; two mAbs, 7B8 and 8G4, were selected for their effective affinity to the antigen of interest and were characterized, demonstrating a promising future diagnostic device to detect *A. fumigatus* galactomannan (Matveev et al., 2018).

### Mycograb

Antibody-based therapies against fungal infections have been developed more and more in these last years, but most of them have been derived from mice. The generation of genetically engineered antibodies in a human configuration is a great innovation that allows also immunocompromised patients to respond to fungal infections, because the cooperation of immune system is not required for the antibodies to function. One of the first examples of a recombinant antibody was Mycograb^®^, a human monoclonal antibody against fungal heat shock protein 90 (HSP90). This protein has two ATP binding sites: when HSP90 binds ATP, a molecular conformational change occurs and Mycograb^®^might be responsible for the inhibition of HSP90 by blocking this change (Matthews et al., 2003). *In vitro* and initial clinical data have demonstrated that Mycograb^®^is active against *Candida* spp. if used alone, but showed therapeutic synergism when combined with amphotericin B, fluconazole, and caspofungin (Bugli et al., 2013). A therapeutic strategy based on the combination of drugs could increase the efficacy and eradicate the problem of antimicotic drug resistance.

### MAb 18B7

In the field of mycology, despite several monoclonal antibodies produced and tested for their efficacy against fungal species, there are very few examples of antibody-based therapies that have been studied clinically. Among these rare cases, a murine antibody against glucuronoxylomannan, a cryptococcal capsular polysaccharide, was produced and named mAb 18B7, tested *in vitro* and in animals, and then translated to a phase I dose-escalation study in HIV-infected patients who had been successfully treated for cryptococcal meningitis. Several doses of mAb 18B7 were used to establish the better treatment, but side effects were noticed and increased with the higher dose of administered drug: this led to defining the well tolerated dose that showed a pharmacological effect, demonstrated by a reduction in the serum cryptococcal antigen titers (Larsen et al., 2005).

In addition to the improvement of classical antifungals, other drugs against new targets have been developed (Table 3). Further strategies still in development examine different antifungal drugs with the aim of using them both in therapeutic and in prophylactic therapy. Vaccines can be prophylactic for immunocompetent subjects who are going to start an immunosuppressive treatment, and therapeutic for already immunocompromised subjects (Nanjappa et al., 2012; Silva et al., 2017).

**Table 3:** New antifungal drugs.

Antifungal drug	Mechanism of action
Aureobasidin A	Inhibition of inositol phosphoryl-ceramide (IPC) synthase (Aeed et al., 2009; Mor et al., 2015; Lazzarini et al., 2018)
APX001	Inhibition of glycosyl phosphatidylinositol (GPI) synthesis (Miyazaki et al., 2011; Wiederhold et al., 2015; Komath et al., 2018)
ASP2397	Disrupting the intracellular fungal biochemical machinery (Nakamura et al., 2017)
MGCD290	Inhibitor of HDAC (histone deacetylase) (Pfaller et al., 2009a, 2015)
FTY720	Modulator of sphingosine-1-phosphate receptor (Hagihara et al., 2013)
AR-12	Downregulation of host chaperones and inhibition of acetyl-CoA synthetase 1 (Koselny et al., 2016)
Nikkomycines	Inhibition of chitin synthase (Hector et al., 1990; Shubitz et al., 2014)

Immunotherapy based on factors stimulating the cytokine granulocitosis require further experimental studies, but some of them, such as GM-CSF, have already shown promising results and are in phase IV clinical trial (Wan et al., 2015).

Other possible antifungal drugs are those of antimicrobial peptides (AMPs) like VL-2397 and PAC-113, which are in phase II clinical trials. However, costs, toxicity, stability, and brief half-life are still the biggest unsolved issues (Nicola et al., 2018).

## MAb 2G8: a Successful Monoclonal Antibody Against β-1,3 Glucan of Pathogenic Fungi

Targeting β-1,3 glucan can be useful in fighting fungal infections, and for this reason the idea of generating a vaccine based on this fungal cell wall component has been taken under consideration for several years. The concept of a unique vaccine for different fungal species is not achievable in practice, because the organization of β-glucans can vary amongst the organisms, determining a difference in the bonds of polysaccharidic structure, and all these dissimilarities result in a varying immunogenic capacity (Masuoka, 2004; Cassone and Torosantucci, 2006). However, β-glucan remains a significant antigen for vaccine development, as it becomes strongly immunogenic against numerous pathogenic fungi if conjugated to a protein carrier. The first glycol-conjugate vaccine (Torosantucci et al., 2005) was generated by extracting laminarin, a β-1,3 glucan from the brown alga *Laminaria digitata*, in order to avoid contamination from other fungal antigens, such as mannoproteins; the low immunogenicity was solved by conjugating laminarin with the genetically detoxified diphtheria toxoid CRM197, a protein carrier already tested in other human vaccines (Giannini et al., 1984; Ho et al., 2000). This vaccine, defined as Lam-CRM, was produced in various preparations and characterized by nuclear magnetic resonance for the integrity of the linked saccharide chains. Successively, it was verified to be protective against systemic candidiasis in mice and vaginal candidiasis in rats. These data indicated the possibility of developing an anti-β-glucan murine monoclonal antibody (mAb) to give passive protection against fungal infections; mAb 2G8 (IgG2b isotype) was generated and showed high affinity for β-1,3 glucan and protection against *Candida albicans* and *Aspergillus fumigatus* (Torosantucci et al., 2005). In particular, *in vitro* experiments demonstrated the ability of Lam-CRM immune serum and mAb 2G8 to strongly inhibit growth of *C. albicans* and hyphal growth of *A. fumigatus*. MAb 2G8 showed its efficacy also *in vivo*: in a systemic mouse model of *Candida* infection, a single intraperitoneal administration of the mAb 2G8 2 h before an intravenous injection with the same fungal dose as the one used for testing passive protection by Lam-CRM immune serum conferred significant protection, (averaging a >1-log decrease in the fungus kidney burden), compared to the control mice treated with an irrelevant anti-CRM mAb. In the same manner, in a rat model of vaginal candidiasis a single intravaginal administration of mAb 2G8 induced a more rapid fungus clearance, with the complete resolution of the infection after 21 days, compared to the control rats treated with an irrelevant anti-CRM mAb (Torosantucci et al., 2005). The results of mAb 2G8 infusion were also verified on *Cryptococcus neoformans*, confirming their capability to bind and inhibit the growth and capsule formation of this fungal species *in vitro*, and demonstrating that mAb 2G8 decreased the size of the capsule in two encapsulated strains, both *in vitro* and *in vivo*. Furthermore, the anti-β-glucan mAb 2G8 induced the death of acapsular *C. neoformans* cells through opsonization, thus increasing the phagocytosis by human monocytes (Rachini et al., 2007).

In a different study performed on *C. albicans*, mAb 2G8 was evaluated in comparison with a murine monoclonal antibody belonging to a different isotype, IgM, but showing the same Complementarity Determining Regions (CDRs) of both light and heavy chain: this antibody, named mAb 1E12, recognized oligosaccharides with β-1,3, β-1,4 and β-1,6 linkages, while mAb 2G8 was specific for β-1,3 glucan. In addition, mAb 2G8 showed significant protection in animal models of candidiasis, whereas mAb 1E12 was merely poorly protective and did not exert any fungal growth-inhibitory activity (Torosantucci et al., 2009). This evaluation concluded that mAb 2G8 remarkably contrasted fungal virulence by specifically binding some β-glucan epitopes that play a crucial role in the cell wall assembly.

## Novel Therapeutic Combination Strategy to Overcome Fungal Resistance

Antifungal resistance is an emerging problem in the treatment of fungal infectious diseases. Recent evidence has demonstrated that the percentage of resistant yeast strains has grown in the last years, due to a widespread use of common antifungal small molecules. Fluconazole is the most common and useful drug, since it presents reduced costs, is well-tolerated and allows oral administration. *Candida* spp., in particular non-*C. albicans* spp., that are responsible for many serious infections, are resistant to fluconazole and this substantially predisposes resistance to other azoles that share the same mechanism of action, inducing fungal defense strategies to impair the biological effect of this class of drugs. In different health care institutions and countries the rate of fluconazole resistance varies, from 12 to 18% for *C. glabrata* in the United States (Pfaller et al., 2009b) reaching 50% in Chinese intensive care organizations also for *C. krusei, C. parapsilosis* and *C. tropicalis* (Guo et al., 2013; Liao et al., 2015; Xiao et al., 2015). Echinocandins resistance is a less frequent mechanism and is mainly correlated to the antifungal treatment of each patient, with a directly proportional ratio between the time of exposure to echinocandins and the development of resistance against these compounds: there is clinical evidence of a 90% treatment failure rate in patients previously treated with echinocandins and infected by resistant strains of *C. glabrata*, successively isolated (Shields et al., 2012, 2013). Very recently, an epidemiological study was conducted on 54 patients with *C. auris* infection from Pakistan, India, South Africa, and Venezuela during the years 2012–2015; through antifungal susceptibility testing and whole-genome sequencing it was demonstrated that 93% of isolates were resistant to fluconazole, 35% to amphotericin B, and 7% to echinocandins; 41% were resistant to 2 antifungal classes and 4% were resistant to three classes (Lockhart et al., 2017). These data are a confirmation of the relevance of growing resistance and the fungal ability to show multidrug resistance, making the choice of the correct therapy for each yeast infection a major clinical challenge. Regarding the resistance in *Aspergillus* spp., in addition to point mutations, the environmental exposure to azoles largely used in agriculture can increase the problem (Wiederhold, 2017).

This evidence and knowledge signifies there is a need for novel medical approaches, new therapeutic strategies that take into account the fungal mechanisms of resistance, targeting specific molecules through different pathways/mechanisms. The choice of the molecular target is important for drug development, and β-glucans are a valid option, due to their abundance in the fungal cell wall of many yeasts spp. A novel class of glucan synthase inhibitors has recently taken on high relevance among antifungal drugs, they present the same mechanism of action exerted by echinocandins, but are structurally different.

MK-3118 (SCY-078) is a semi-synthetic derivative of the natural product enfumafungin, a potent inhibitor of fungal β-1,3-D-glucan synthases; chemically it is a triterpenoid and the great advantage is the oral bioavailability of this compound, an important step forward in antifungal drug administration methods, since most of the currently used drugs have only intravenous formulations. This compound is powerful because it is active against both wild-type and antifungal-resistant strains of *Aspergillus* spp.; a total of 71 *Aspergillus* strains were evaluated to establish the minimum effective concentration (MEC) endpoints for MK-3118 activity, because the mechanism of action based on inhibition of the fungal enzyme β-1,3 glucan synthase determines a reduced hyphal extension of *Aspergillus* spp. and not a complete growth arrest (Pfaller et al., 2013). MK-3118 was also tested against a collection of 135 selected clinical isolates representing the most clinically relevant non-*Aspergillus* fungal pathogens, including *Rhizopus* spp., *Mucor* spp., *Rhizomucor* spp., *Cunninghamella* spp., *Lichtheimia* spp. (previously *Absidia* spp.), *Fusarium* spp., *Scedosporium apiospermum*/*Pseudallescheria boydii* complex, *Scedosporium prolificans, Purpureocillium lilacinum* (previously *Paecilomyces lilacinus*), *Paecilomyces variotii*, and *Scopulariopsis* spp. In addition to the antifungal activity similar to that of echinocandins, MK-3118 was the only compound that showed activity against *S. prolificans* and *P. variotii*, for which there is a great lack of valid therapeutic possibilities (Schell et al., 2017). Through broth microdilution, antifungal carryover, and time-kill dynamics, the activity of MK-3118 was verified with positive results, against *C. albicans*, C. *parapsilosis, C. tropicalis* (Scorneaux et al., 2017) and on many *fks*-mediated echinocandin-resistant strains, in particular those belonging to *C. albicans* and *C. glabrata* (Jimenez-Ortigosa et al., 2014).

The efficacy of this novel antifungal drug was also investigated in a neutropenic murine model of invasive candidiasis, through a pharmacodynamic evaluation that showed very similar results to those for echinocandin intravenous formulations, even if the study presented a limited number of isolates utilized (Lepak et al., 2015).

A very recent study analyzed the antifungal activity of MK-3118 in combination with other antifungal agents in the treatment of invasive aspergillosis: the synergistic effect of MK-3118 and amphotericin B was exerted both against wild-type and *cyp51* mutant *A. fumigatus* strains, suggesting that the combination of drugs could be particularly powerful in cases with a suspected azole resistance (Ghannoum et al., 2018).

The combination of drugs could be the successful approach to fighting fungal infections, reducing the possibility of treatment failure due to an adaptive fungal response, very frequent principally in long-term therapies. Since β-1,3-D-glucan has been verified as an ideal molecular target for antifungal drugs, combining two agents that target this essential component of the fungal cell wall could be a novel and interesting therapeutic strategy. The hypothesis of investigating the synergistic antifungal effect of MK-3118 together with those well described for mAb 2G8 could be an innovative approach. The option to use a glucan synthase inhibitor along with a monoclonal antibody that directly inhibits β-1,3-D-glucan presents a dual mechanism with antifungal activity, which acts through different mechanisms to solve the problem of resistance development. In addition to the *in vitro* experiments performed to confirm antifungal activity of these two compounds, preclinical and clinical evaluations are necessary to validate this combination of drugs as a new antifungal treatment. In reference to the well-known ability of these agents to work singly, we expect a synergy in the combined functioning, to fight the most common fungal infections and ensure protection against resistance mechanisms.

## Conclusion

Despite the existence of a number of therapeutic opportunities, fungal infections remain amongst the diseases with the most urgent and widespread medical need. The underlying motivation is the limited success of antifungal drugs, which can be mostly attributed to delays in the diagnosis and detection of fungal infection. The present challenge is to identify targets for early diagnosis, to allow clinicians to treat patients as promptly as possible. The necessity for novel fungal molecular targets that can be directly recognized and strongly inhibited by new antifungal drugs, remains the crucial point for fungal infectious diseases. As concerns the improvement of currently used antifungal molecules, the method of administration, bioavailability and toxicity are good points for discussion, but the resistance of fungi strains continues to be the most serious problem connected to antifungal drug treatments. In closing, antifungal vaccines for the prevention of invasive fungal diseases may well be a good line of defense against the infections and the progression of related diseases, especially for patients with a high risk factor and the predisposition for this category of infectious diseases.

## Author Contributions

TDM and IG wrote the manuscript. LA critically revised it. MM and EM supervised the work.

## Conflict of Interest Statement

IG, LA, and EM are employees at Takis s.r.l. MM is the scientific director of Diatheva s.r.l., and holds shares in the company. TDM was partially supported by a Diatheva grant. Both companies are involved in mAb 2G8 research program.

## Footnotes

1 http://www.drugbank.ca

## References

[B1] Aeed P. A. Young C. L. Nagiec M. M. Elhammer A. P. (2009). Inhibition of inositol phosphorylceramide synthase by the cyclic peptide aureobasidin A. *Antimicrob. Agents Chemother.* 53 496–504. 10.1128/AAC.00633-08 19047657 PMC2630602

[B2] Alcazar-Fuoli L. Mellado E. (2012). Ergosterol biosynthesis in Aspergillus fumigatus: its relevance as an antifungal target and role in antifungal drug resistance. *Front. Microbiol.* 3:439. 10.3389/fmicb.2012.00439 23335918 PMC3541703

[B3] Alexander B. D. Johnson M. D. Pfeiffer C. D. Jimenez-Ortigosa C. Catania J. Booker R. (2013). Increasing echinocandin resistance in Candida glabrata: clinical failure correlates with presence of FKS mutations and elevated minimum inhibitory concentrations. *Clin. Infect. Dis.* 56 1724–1732. 10.1093/cid/cit136 23487382 PMC3658363

[B4] Andes D. R. Safdar N. Baddley J. W. Playford G. Reboli A. C. Rex J. H. (2012). Impact of treatment strategy on outcomes in patients with candidemia and other forms of invasive candidiasis: a patient-level quantitative review of randomized trials. *Clin. Infect. Dis.* 54 1110–1122. 10.1093/cid/cis021 22412055

[B5] Baldin C. Valiante V. Kruger T. Schafferer L. Haas H. Kniemeyer O. (2015). Comparative proteomics of a tor inducible *Aspergillus fumigatus* mutant reveals involvement of the Tor kinase in iron regulation. *Proteomics* 15 2230–2243. 10.1002/pmic.201400584 25728394

[B6] Bard M. Lees N. D. Turi T. Craft D. Cofrin L. Barbuch R. (1993). Sterol synthesis and viability of erg11 (cytochrome P450 lanosterol demethylase) mutations in *Saccharomyces cerevisiae* and *Candida albicans*. *Lipids* 28 963–967. 10.1007/BF02537115 8277826

[B7] Bratton E. W. El Husseini N. Chastain C. A. Lee M. S. Poole C. Sturmer T. (2012). Comparison and temporal trends of three groups with cryptococcosis: HIV-infected, solid organ transplant, and HIV-negative/non-transplant. *PLoS One* 7:e43582. 10.1371/journal.pone.0043582 22937064 PMC3427358

[B8] Brena S. Cabezas-Olcoz J. Moragues M. D. Fernandez De Larrinoa I. Dominguez A. (2011). Fungicidal monoclonal antibody C7 interferes with iron acquisition in *Candida albicans*. *Antimicrob. Agents Chemother.* 55 3156–3163. 10.1128/AAC.00892-10 21518848 PMC3122412

[B9] Bugli F. Cacaci M. Martini C. Torelli R. Posteraro B. Sanguinetti M. (2013). Human monoclonal antibody-based therapy in the treatment of invasive candidiasis. *Clin. Dev. Immunol.* 2013:403121. 10.1155/2013/403121 23878583 PMC3710647

[B10] Carter P. J. Lazar G. A. (2018). Next generation antibody drugs: pursuit of the ‘high-hanging fruit’. *Nat. Rev. Drug Discov.* 17 197–223. 10.1038/nrd.2017.227 29192287

[B11] Cassone A. Torosantucci A. (2006). Opportunistic fungi and fungal infections: the challenge of a single, general antifungal vaccine. *Expert Rev. Vaccines* 5 859–867. 10.1586/14760584.5.6.859 17184223

[B12] Chaturvedi A. K. Kavishwar A. Shiva Keshava G. B. Shukla P. K. (2005). Monoclonal immunoglobulin G1 directed against *Aspergillus fumigatus* cell wall glycoprotein protects against experimental murine aspergillosis. *Clin. Diagn. Lab. Immunol.* 12 1063–1068. 10.1128/CDLI.12.9.1063-1068.2005 16148172 PMC1235786

[B13] Clavero-Alvarez A. Di Mambro T. Perez-Gaviro S. Magnani M. Bruscolini P. (2018). Humanization of antibodies using a statistical inference approach. *Sci. Rep.* 8:14820. 10.1038/s41598-018-32986-y 30287940 PMC6172228

[B14] Dadachova E. Bryan R. A. Apostolidis C. Morgenstern A. Zhang T. Moadel T. (2006). Interaction of radiolabeled antibodies with fungal cells and components of the immune system in vitro and during radioimmunotherapy for experimental fungal infection. *J. Infect. Dis.* 193 1427–1436. 10.1086/503369 16619191

[B15] Dadachova E. Burns T. Bryan R. A. Apostolidis C. Brechbiel M. W. Nosanchuk J. D. (2004). Feasibility of radioimmunotherapy of experimental pneumococcal infection. *Antimicrob. Agents Chemother.* 48 1624–1629. 10.1128/AAC.48.5.1624-1629.2004 15105113 PMC400592

[B16] Dadachova E. Nakouzi A. Bryan R. A. Casadevall A. (2003). Ionizing radiation delivered by specific antibody is therapeutic against a fungal infection. *Proc. Natl. Acad. Sci. U.S.A.* 100 10942–10947. 10.1073/pnas.1731272100 12930899 PMC196907

[B17] Delmas G. Park S. Chen Z. W. Tan F. Kashiwazaki R. Zarif L. (2002). Efficacy of orally delivered cochleates containing amphotericin B in a murine model of aspergillosis. *Antimicrob. Agents Chemother.* 46 2704–2707. 10.1128/AAC.46.8.2704-2707.2002 12121962 PMC127382

[B18] Dick J. D. Merz W. G. Saral R. (1980). Incidence of polyene-resistant yeasts recovered from clinical specimens. *Antimicrob. Agents Chemother.* 18 158–163. 10.1128/AAC.18.1.158 7416742 PMC283956

[B19] Douglas C. M. (2001). Fungal beta(1,3)-D-glucan synthesis. *Med. Mycol.* 39(Suppl. 1) 55–66. 10.1080/mmy.39.1.55.66 11800269

[B20] Douglas L. M. Konopka J. B. (2014). Fungal membrane organization: the eisosome concept. *Annu. Rev. Microbiol.* 68 377–393. 10.1146/annurev-micro-091313-103507 25002088

[B21] Douglas L. M. Konopka J. B. (2016). Plasma membrane organization promotes virulence of the human fungal pathogen Candida albicans. *J. Microbiol.* 54 178–191. 10.1007/s12275-016-5621-y 26920878 PMC5650914

[B22] Du Pre S. Beckmann N. Almeida M. C. Sibley G. E. M. Law D. Brand A. C. (2018). Effect of the novel antifungal drug f901318 (olorofim) on growth and viability of *Aspergillus fumigatus*. *Antimicrob. Agents Chemother.* 62 e231–18. 10.1128/AAC.00231-18 29891595 PMC6105813

[B23] Edlind T. D. Katiyar S. K. (2010). Mutational analysis of flucytosine resistance in Candida glabrata. *Antimicrob. Agents Chemother.* 54 4733–4738. 10.1128/AAC.00605-10 20823283 PMC2976130

[B24] Eggimann P. Garbino J. Pittet D. (2003). Epidemiology of Candida species infections in critically ill non-immunosuppressed patients. *Lancet Infect. Dis.* 3 685–702. 10.1016/S1473-3099(03)00801-6 14592598

[B25] Enoch D. A. Ludlam H. A. Brown N. M. (2006). Invasive fungal infections: a review of epidemiology and management options. *J. Med. Microbiol.* 55 809–818. 10.1099/jmm.0.46548-0 16772406

[B26] Garbino J. Kolarova L. Rohner P. Lew D. Pichna P. Pittet D. (2002). Secular trends of candidemia over 12 years in adult patients at a tertiary care hospital. *Medicine* 81 425–433. 10.1097/00005792-200211000-00003 12441899

[B27] Gaughan C. L. (2016). The present state of the art in expression, production and characterization of monoclonal antibodies. *Mol. Divers* 20 255–270. 10.1007/s11030-015-9625-z 26299798

[B28] Gebremariam T. Wiederhold N. P. Fothergill A. W. Garvey E. P. Hoekstra W. J. Schotzinger R. J. (2015). VT-1161 Protects Immunosuppressed Mice from Rhizopus arrhizus var. Arrhizus Infection. *Antimicrob. Agents Chemother.* 59 7815–7817. 10.1128/AAC.01437-15 26369977 PMC4649228

[B29] Ghannoum M. Long L. Larkin E. L. Isham N. Sherif R. Borroto-Esoda K. (2018). Evaluation of the antifungal activity of the novel oral glucan synthase inhibitor SCY-078, singly and in combination, for the treatment of invasive aspergillosis. *Antimicrob. Agents Chemother.* 62 e244–18. 10.1128/AAC.00244-18 29610204 PMC5971594

[B30] Ghannoum M. A. Rice L. B. (1999). Antifungal agents: mode of action, mechanisms of resistance, and correlation of these mechanisms with bacterial resistance. *Clin. Microbiol. Rev.* 12 501–517. 10.1128/CMR.12.4.501 10515900 PMC88922

[B31] Giannini G. Rappuoli R. Ratti G. (1984). The amino-acid sequence of two non-toxic mutants of diphtheria toxin: CRM45 and CRM197. *Nucleic Acids Res.* 12 4063–4069. 10.1093/nar/12.10.4063 6427753 PMC318816

[B32] Gow N. A. R. Latge J. P. Munro C. A. (2017). The fungal cell wall: structure, biosynthesis, and function. *Microbiol. Spectr.* 5. 10.1128/microbiolspec.FUNK-0035-2016 28513415 PMC11687499

[B33] Grosse C. Heinekamp T. Kniemeyer O. Gehrke A. Brakhage A. A. (2008). Protein kinase A regulates growth, sporulation, and pigment formation in *Aspergillus fumigatus*. *Appl. Environ. Microbiol.* 74 4923–4933. 10.1128/AEM.00470-08 18539819 PMC2519360

[B34] Grossmann G. Malinsky J. Stahlschmidt W. Loibl M. Weig-Meckl I. Frommer W. B. (2008). Plasma membrane microdomains regulate turnover of transport proteins in yeast. *J. Cell. Biol.* 183 1075–1088. 10.1083/jcb.200806035 19064668 PMC2600745

[B35] Grossmann G. Opekarova M. Malinsky J. Weig-Meckl I. Tanner W. (2007). Membrane potential governs lateral segregation of plasma membrane proteins and lipids in yeast. *EMBO J.* 26 1–8. 10.1038/sj.emboj.7601466 17170709 PMC1782361

[B36] Guo F. Yang Y. Kang Y. Zang B. Cui W. Qin B. (2013). Invasive candidiasis in intensive care units in China: a multicentre prospective observational study. *J. Antimicrob. Chemother.* 68 1660–1668. 10.1093/jac/dkt083 23543609

[B37] Hagihara K. Kita A. Mizukura A. Yao M. Kitai Y. Kunoh T. (2013). Fingolimod (FTY720) stimulates Ca(2+)/calcineurin signaling in fission yeast. *PLoS One* 8:e81907. 10.1371/journal.pone.0081907 24312601 PMC3849299

[B38] Hector R. F. Zimmer B. L. Pappagianis D. (1990). Evaluation of nikkomycins X and Z in murine models of coccidioidomycosis, histoplasmosis, and blastomycosis. *Antimicrob. Agents Chemother.* 34 587–593. 10.1128/AAC.34.4.587 2344165 PMC171648

[B39] Ho M. M. Bolgiano B. Corbel M. J. (2000). Assessment of the stability and immunogenicity of meningococcal oligosaccharide C-CRM197 conjugate vaccines. *Vaccine* 19 716–725. 10.1016/S0264-410X(00)00261-9 11115692

[B40] Hobson R. P. (2003). The global epidemiology of invasive Candida infections–is the tide turning? *J. Hosp. Infect.* 55 159–168. 10.1016/j.jhin.2003.08.012 14572481

[B41] Husain S. Alexander B. D. Munoz P. Avery R. K. Houston S. Pruett T. (2003). Opportunistic mycelial fungal infections in organ transplant recipients: emerging importance of non-aspergillus mycelial fungi. *Clin. Infect. Dis.* 37 221–229. 10.1086/375822 12856215

[B42] Jimenez-Ortigosa C. Paderu P. Motyl M. R. Perlin D. S. (2014). Enfumafungin derivative MK-3118 shows increased in vitro potency against clinical echinocandin-resistant candida species and aspergillus species isolates. *Antimicrob. Agents Chemother.* 58 1248–1251. 10.1128/AAC.02145-13 24323472 PMC3910825

[B43] Kaplon H. Reichert J. M. (2018). Antibodies to watch in 2018. *MAbs* 10 183–203. 10.1080/19420862.2018.1415671 29300693 PMC5825203

[B44] Karyotakis N. C. Anaissie E. J. Hachem R. Dignani M. C. Samonis G. (1993). Comparison of the efficacy of polyenes and triazoles against hematogenous *Candida krusei* infection in neutropenic mice. *J. Infect. Dis.* 168 1311–1313. 10.1093/infdis/168.5.1311 8228370

[B45] Kimura L. H. Pearsall N. N. (1980). Relationship between germination of Candida albicans and increased adherence to human buccal epithelial cells. *Infect. Immun.* 28 464–468. 6995309 10.1128/iai.28.2.464-468.1980 PMC550958

[B46] Komath S. S. Singh S. L. Pratyusha V. A. Sah S. K. (2018). Generating anchors only to lose them: the unusual story of glycosylphosphatidylinositol anchor biosynthesis and remodeling in yeast and fungi. *IUBMB Life* 70 355–383. 10.1002/iub.1734 29679465

[B47] Kontoyiannis D. P. Lewis R. E. (2002). Antifungal drug resistance of pathogenic fungi. *Lancet* 359 1135–1144. 10.1016/S0140-6736(02)08162-X 11943280

[B48] Koselny K. Green J. Didone L. Halterman J. P. Fothergill A. W. Wiederhold N. P. (2016). The celecoxib derivative AR-12 has broad-spectrum antifungal activity in vitro and improves the activity of fluconazole in a murine model of cryptococcosis. *Antimicrob. Agents Chemother.* 60 7115–7127. 10.1128/AAC.01061-16 27645246 PMC5118990

[B49] Larsen R. A. Pappas P. G. Perfect J. Aberg J. A. Casadevall A. Cloud G. A. (2005). Phase I evaluation of the safety and pharmacokinetics of murine-derived anticryptococcal antibody 18B7 in subjects with treated cryptococcal meningitis. *Antimicrob. Agents Chemother.* 49 952–958. 10.1128/AAC.49.3.952-958.2005 15728888 PMC549259

[B50] Lazzarini C. Haranahalli K. Rieger R. Ananthula H. K. Desai P. B. Ashbaugh A. (2018). Acylhydrazones as antifungal agents targeting the synthesis of fungal sphingolipids. *Antimicrob. Agents Chemother.* 62 e156–18. 10.1128/AAC.00156-18 29507066 PMC5923120

[B51] Lepak A. J. Marchillo K. Andes D. R. (2015). Pharmacodynamic target evaluation of a novel oral glucan synthase inhibitor, SCY-078 (MK-3118), using an in vivo murine invasive candidiasis model. *Antimicrob. Agents Chemother.* 59 1265–1272. 10.1128/AAC.04445-14 25512406 PMC4335824

[B52] Levin D. E. (2011). Regulation of cell wall biogenesis in Saccharomyces cerevisiae: the cell wall integrity signaling pathway. *Genetics* 189 1145–1175. 10.1534/genetics.111.128264 22174182 PMC3241422

[B53] Liao X. Qiu H. Li R. Guo F. Liu W. Kang M. (2015). Risk factors for fluconazole-resistant invasive candidiasis in intensive care unit patients: an analysis from the china survey of candidiasis study. *J. Crit. Care* 30 e861–e865. 10.1016/j.jcrc.2015.04.002 26002430

[B54] Lockhart S. R. Etienne K. A. Vallabhaneni S. Farooqi J. Chowdhary A. Govender N. P. (2017). Simultaneous emergence of multidrug-resistant candida auris on 3 continents confirmed by whole-genome sequencing and epidemiological analyses. *Clin. Infect. Dis.* 64 134–140. 10.1093/cid/ciw691 27988485 PMC5215215

[B55] Lockhart S. R. Fothergill A. W. Iqbal N. Bolden C. B. Grossman N. T. Garvey E. P. (2016). The investigational fungal Cyp51 inhibitor VT-1129 demonstrates potent in vitro activity against cryptococcus neoformans and cryptococcus gattii. *Antimicrob. Agents Chemother.* 60 2528–2531. 10.1128/AAC.02770-15 26787697 PMC4808209

[B56] Lonberg N. (2008). Human monoclonal antibodies from transgenic mice. *Handb. Exp. Pharmacol.* 181 69–97. 10.1007/978-3-540-73259-4_4 18071942 PMC7120671

[B57] Malinsky J. Opekarova M. Tanner W. (2010). The lateral compartmentation of the yeast plasma membrane. *Yeast* 27 473–478. 10.1002/yea.1772 20641012

[B58] Marr K. A. Carter R. A. Crippa F. Wald A. Corey L. (2002). Epidemiology and outcome of mould infections in hematopoietic stem cell transplant recipients. *Clin. Infect. Dis.* 34 909–917. 10.1086/339202 11880955

[B59] Marr K. A. Schlamm H. T. Herbrecht R. Rottinghaus S. T. Bow E. J. Cornely O. A. (2015). Combination antifungal therapy for invasive aspergillosis: a randomized trial. *Ann. Intern. Med.* 162 81–89. 10.7326/M13-2508 25599346

[B60] Martin S. G. Arkowitz R. A. (2014). Cell polarization in budding and fission yeasts. *FEMS Microbiol. Rev.* 38 228–253. 10.1111/1574-6976.12055 24354645

[B61] Masuoka J. (2004). Surface glycans of Candida albicans and other pathogenic fungi: physiological roles, clinical uses, and experimental challenges. *Clin. Microbiol. Rev.* 17 281–310. 10.1128/CMR.17.2.281-310.2004 15084502 PMC387410

[B62] Matthews R. C. Rigg G. Hodgetts S. Carter T. Chapman C. Gregory C. (2003). Preclinical assessment of the efficacy of mycograb, a human recombinant antibody against fungal HSP90. *Antimicrob. Agents Chemother.* 47 2208–2216. 10.1128/AAC.47.7.2208-2216.2003 12821470 PMC161838

[B63] Matveev A. L. Krylov V. B. Emelyanova L. A. Solovev A. S. Khlusevich Y. A. Baykov I. K. (2018). Novel mouse monoclonal antibodies specifically recognize *Aspergillus fumigatus* galactomannan. *PLoS One* 13:e0193938. 10.1371/journal.pone.0193938 29518144 PMC5843280

[B64] Miyazaki M. Horii T. Hata K. Watanabe N. A. Nakamoto K. Tanaka K. (2011). In vitro activity of E1210, a novel antifungal, against clinically important yeasts and molds. *Antimicrob. Agents Chemother.* 55 4652–4658. 10.1128/AAC.00291-11 21825291 PMC3186989

[B65] Mor V. Rella A. Farnoud A. M. Singh A. Munshi M. Bryan A. (2015). Identification of a new class of antifungals targeting the synthesis of fungal sphingolipids. *MBio* 6 e00647–15. 10.1128/mBio.00647-15 26106079 PMC4479701

[B66] Moragues M. D. Omaetxebarria M. J. Elguezabal N. Sevilla M. J. Conti S. Polonelli L. (2003). A monoclonal antibody directed against a Candida albicans cell wall mannoprotein exerts three anti-C. *albicans activities*. *Infect. Immun.* 71 5273–5279. 10.1128/IAI.71.9.5273-5279.2003 12933874 PMC187351

[B67] Murphy E. R. Kim K. T. (2012). Insights into eisosome assembly and organization. *J. Biosci.* 37 295–500. 10.1007/s12038-012-9206-6 22581335

[B68] Nakamura I. Yoshimura S. Masaki T. Takase S. Ohsumi K. Hashimoto M. (2017). ASP2397: a novel antifungal agent produced by acremonium persicinum MF-347833. *J. Antibiot.* 70 45–51. 10.1038/ja.2016.107 27599768

[B69] Nanjappa S. G. Heninger E. Wuthrich M. Gasper D. J. Klein B. S. (2012). Tc17 cells mediate vaccine immunity against lethal fungal pneumonia in immune deficient hosts lacking CD4+ T cells. *PLoS Pathog* 8:e1002771. 10.1371/journal.ppat.1002771 22829762 PMC3400565

[B70] Nicola A. M. Albuquerque P. Paes H. C. Fernandes L. Costa F. F. Kioshima E. S. (2018). Antifungal drugs: new insights in research & development. *Pharmacol. Ther.* 10.1016/j.pharmthera.2018.10.008 [Epub ahead of print]. 30347212

[B71] Nyazika T. K. Hagen F. Machiridza T. Kutepa M. Masanganise F. Hendrickx M. (2016). Cryptococcus neoformans population diversity and clinical outcomes of HIV-associated cryptococcal meningitis patients in zimbabwe. *J. Med. Microbiol.* 65 1281–1288. 10.1099/jmm.0.000354 27638836

[B72] Oliver J. D. Sibley G. E. M. Beckmann N. Dobb K. S. Slater M. J. Mcentee L. (2016). F901318 represents a novel class of antifungal drug that inhibits dihydroorotate dehydrogenase. *Proc. Natl. Acad. Sci. U.S.A.* 113 12809–12814. 10.1073/pnas.1608304113 27791100 PMC5111691

[B73] Olivera-Couto A. Grana M. Harispe L. Aguilar P. S. (2011). The eisosome core is composed of BAR domain proteins. *Mol. Biol. Cell.* 22 2360–2372. 10.1091/mbc.E10-12-1021 21593205 PMC3128537

[B74] Ong V. Hough G. Schlosser M. Bartizal K. Balkovec J. M. James K. D. (2016). Preclinical Evaluation of the stability, safety, and efficacy of CD101, a Novel Echinocandin. *Antimicrob. Agents Chemother.* 60 6872–6879. 10.1128/AAC.00701-16 27620474 PMC5075098

[B75] Perfect J. R. (2017). The antifungal pipeline: a reality check. *Nat. Rev. Drug Discov.* 16 603–616. 10.1038/nrd.2017.46 28496146 PMC5760994

[B76] Pfaller M. A. Messer S. A. Georgopapadakou N. Martell L. A. Besterman J. M. Diekema D. J. (2009a). Activity of MGCD290, a Hos2 histone deacetylase inhibitor, in combination with azole antifungals against opportunistic fungal pathogens. *J. Clin. Microbiol.* 47 3797–3804. 10.1128/JCM.00618-09 19794038 PMC2786684

[B77] Pfaller M. A. Messer S. A. Hollis R. J. Boyken L. Tendolkar S. Kroeger J. (2009b). Variation in susceptibility of bloodstream isolates of *Candida glabrata* to fluconazole according to patient age and geographic location in the United States in 2001 to 2007. *J. Clin. Microbiol.* 47 3185–3190. 10.1128/JCM.00946-09 19656983 PMC2756923

[B78] Pfaller M. A. Messer S. A. Motyl M. R. Jones R. N. Castanheira M. (2013). In vitro activity of a new oral glucan synthase inhibitor (MK-3118) tested against aspergillus spp. by CLSI and EUCAST broth microdilution methods. *Antimicrob. Agents Chemother.* 57 1065–1068. 10.1128/AAC.01588-12 23229479 PMC3553681

[B79] Pfaller M. A. Messer S. A. Rhomberg P. R. Jones R. N. Castanheira M. (2016). Activity of a long-acting echinocandin, CD101, determined using CLSI and EUCAST reference methods, against candida and aspergillus spp., including echinocandin- and azole-resistant isolates. *J. Antimicrob. Chemother.* 71 2868–2873. 10.1093/jac/dkw214 27287236 PMC5031917

[B80] Pfaller M. A. Rhomberg P. R. Messer S. A. Castanheira M. (2015). In vitro activity of a Hos2 deacetylase inhibitor, MGCD290, in combination with echinocandins against echinocandin-resistant candida species. *Diagn. Microbiol. Infect. Dis.* 81 259–263. 10.1016/j.diagmicrobio.2014.11.008 25600842

[B81] Rachini A. Pietrella D. Lupo P. Torosantucci A. Chiani P. Bromuro C. (2007). An anti-beta-glucan monoclonal antibody inhibits growth and capsule formation of Cryptococcus neoformans in vitro and exerts therapeutic, anticryptococcal activity in vivo. *Infect. Immun.* 75 5085–5094. 10.1128/IAI.00278-07 17606600 PMC2168274

[B82] Reichert J. M. (2017). Antibodies to watch in 2017. *MAbs* 9 167–181. 10.1080/19420862.2016.1269580 27960628 PMC5297518

[B83] Richardson M. D. (2005). Changing patterns and trends in systemic fungal infections. *J. Antimicrob. Chemother.* 56(Suppl. 1) i5–i11. 10.1093/jac/dki218 16120635

[B84] Rispail N. Soanes D. M. Ant C. Czajkowski R. Grunler A. Huguet R. (2009). Comparative genomics of MAP kinase and calcium-calcineurin signalling components in plant and human pathogenic fungi. *Fungal Genet. Biol.* 46 287–298. 10.1016/j.fgb.2009.01.002 19570501

[B85] Roncero C. (2002). The genetic complexity of chitin synthesis in fungi. *Curr. Genet.* 41 367–378. 10.1007/s00294-002-0318-7 12228806

[B86] San Millan R. Elguezabal N. Regulez P. Moragues M. D. Quindos G. Ponton J. (2000). Effect of salivary secretory IgA on the adhesion of Candida albicans to polystyrene. *Microbiology* 146(Pt 9) 2105–2112. 10.1099/00221287-146-9-2105 10974098

[B87] Schell W. A. Jones A. M. Borroto-Esoda K. Alexander B. D. (2017). Antifungal activity of SCY-078 and standard antifungal agents against 178 clinical isolates of resistant and susceptible candida species. *Antimicrob. Agents Chemother.* 61 e1102–e1117. 10.1128/AAC.01102-17 28827419 PMC5655100

[B88] Schuberth C. Wedlich-Soldner R. (2015). Building a patchwork. The yeast plasma membrane as model to study lateral domain formation. *Biochim. Biophys. Acta* 1853 767–774. 10.1016/j.bbamcr.2014.12.019 25541280

[B89] Scorneaux B. Angulo D. Borroto-Esoda K. Ghannoum M. Peel M. Wring S. (2017). SCY-078 is fungicidal against candida species in time-kill studies. *Antimicrob. Agents Chemother.* 61 e1961–16. 10.1128/AAC.01961-16 28069658 PMC5328566

[B90] Sevilla M. J. Robledo B. Rementeria A. Moragues M. D. Ponton J. (2006). A fungicidal monoclonal antibody protects against murine invasive candidiasis. *Infect. Immun.* 74 3042–3045. 10.1128/IAI.74.5.3042-3045.2006 16622248 PMC1459740

[B91] Shields R. K. Nguyen M. H. Press E. G. Kwa A. L. Cheng S. Du C. (2012). The presence of an FKS mutation rather than MIC is an independent risk factor for failure of echinocandin therapy among patients with invasive candidiasis due to *Candida glabrata*. *Antimicrob. Agents Chemother.* 56 4862–4869. 10.1128/AAC.00027-12 22751546 PMC3421882

[B92] Shields R. K. Nguyen M. H. Press E. G. Updike C. L. Clancy C. J. (2013). Anidulafungin and micafungin MIC breakpoints are superior to that of caspofungin for identifying FKS mutant *Candida glabrata* strains and echinocandin resistance. *Antimicrob. Agents Chemother.* 57 6361–6365. 10.1128/AAC.01451-13 24060873 PMC3837909

[B93] Shubitz L. F. Trinh H. T. Perrill R. H. Thompson C. M. Hanan N. J. Galgiani J. N. (2014). Modeling nikkomycin Z dosing and pharmacology in murine pulmonary coccidioidomycosis preparatory to phase 2 clinical trials. *J. Infect. Dis.* 209 1949–1954. 10.1093/infdis/jiu029 24421256 PMC4038145

[B94] Silva L. B. R. Dias L. S. Rittner G. M. G. Munoz J. E. Souza A. C. O. Nosanchuk J. D. (2017). Dendritic cells primed with paracoccidioides brasiliensis peptide P10 are therapeutic in immunosuppressed mice with paracoccidioidomycosis. *Front. Microbiol.* 8:1057. 10.3389/fmicb.2017.01057 28659882 PMC5469887

[B95] Sui X. Yan L. Jiang Y. Y. (2017). The vaccines and antibodies associated with Als3p for treatment of Candida albicans infections. *Vaccine* 35 5786–5793. 10.1016/j.vaccine.2017.08.082 28911903

[B96] Torosantucci A. Bromuro C. Chiani P. De Bernardis F. Berti F. Galli C. (2005). A novel glyco-conjugate vaccine against fungal pathogens. *J. Exp. Med.* 202 597–606. 10.1084/jem.20050749 16147975 PMC2212864

[B97] Torosantucci A. Chiani P. Bromuro C. De Bernardis F. Palma A. S. Liu Y. (2009). Protection by anti-beta-glucan antibodies is associated with restricted beta-1,3 glucan binding specificity and inhibition of fungal growth and adherence. *PLoS One* 4:e5392. 10.1371/journal.pone.0005392 19399183 PMC2670538

[B98] Valiante V. Macheleidt J. Foge M. Brakhage A. A. (2015). The Aspergillus fumigatus cell wall integrity signaling pathway: drug target, compensatory pathways, and virulence. *Front. Microbiol.* 6:325. 10.3389/fmicb.2015.00325 25932027 PMC4399325

[B99] Vanden Bossche H. Marichal P. Odds F. C. (1994). Molecular mechanisms of drug resistance in fungi. *Trends Microbiol.* 2 393–400. 10.1016/0966-842X(94)90618-1 7850208

[B100] Walker S. S. Xu Y. Triantafyllou I. Waldman M. F. Mendrick C. Brown N. (2011). Discovery of a novel class of orally active antifungal beta-1,3-D-glucan synthase inhibitors. *Antimicrob. Agents Chemother.* 55 5099–5106. 10.1128/AAC.00432-11 21844320 PMC3195001

[B101] Walther T. C. Brickner J. H. Aguilar P. S. Bernales S. Pantoja C. Walter P. (2006). Eisosomes mark static sites of endocytosis. *Nature* 439 998–1003. 10.1038/nature04472 16496001

[B102] Wan L. Zhang Y. Lai Y. Jiang M. Song Y. Zhou J. (2015). Effect of granulocyte-macrophage colony-stimulating factor on prevention and treatment of invasive fungal disease in recipients of allogeneic stem-cell transplantation: a prospective multicenter randomized phase IV trial. *J. Clin. Oncol.* 33 3999–4006. 10.1200/JCO.2014.60.5121 26392095

[B103] Warrilow A. G. Hull C. M. Parker J. E. Garvey E. P. Hoekstra W. J. Moore W. R. (2014). The clinical candidate VT-1161 is a highly potent inhibitor of *Candida albicans* CYP51 but fails to bind the human enzyme. *Antimicrob. Agents Chemother.* 58 7121–7127. 10.1128/AAC.03707-14 25224009 PMC4249504

[B104] White T. C. Marr K. A. Bowden R. A. (1998). Clinical, cellular, and molecular factors that contribute to antifungal drug resistance. *Clin. Microbiol. Rev.* 11 382–402. 10.1128/CMR.11.2.382 9564569 PMC106838

[B105] Wiederhold N. P. (2017). Antifungal resistance: current trends and future strategies to combat. *Infect. Drug Resist.* 10 249–259. 10.2147/IDR.S124918 28919789 PMC5587015

[B106] Wiederhold N. P. Najvar L. K. Fothergill A. W. Mccarthy D. I. Bocanegra R. Olivo M. (2015). The investigational agent E1210 is effective in treatment of experimental invasive candidiasis caused by resistant Candida albicans. *Antimicrob. Agents Chemother.* 59 690–692. 10.1128/AAC.03944-14 25331706 PMC4291422

[B107] Xiao M. Fan X. Chen S. C. Wang H. Sun Z. Y. Liao K. (2015). Antifungal susceptibilities of *Candida glabrata* species complex, *Candida krusei, Candida parapsilosis* species complex and *Candida tropicalis* causing invasive candidiasis in China: 3 year national surveillance. *J. Antimicrob. Chemother.* 70 802–810. 10.1093/jac/dku460 25473027

